# The effects of pharmaceutical interventions on potentially inappropriate medications in older patients: a systematic review and meta-analysis

**DOI:** 10.3389/fpubh.2023.1154048

**Published:** 2023-07-11

**Authors:** Shuang Zhou, Rui Li, Xiaolin Zhang, Yutong Zong, Lili Lei, Zhenhui Tao, Minxue Sun, Hua Liu, Ying Zhou, Yimin Cui

**Affiliations:** ^1^Department of Pharmacy, Peking University First Hospital, Beijing, China; ^2^Department of Pharmaceutical Administration and Clinical Pharmacy, School of Pharmaceutical Science, Peking University, Beijing, China; ^3^Department of Pharmacy, Aerospace Center Hospital, Beijing, China; ^4^Department of Geriatrics, Peking University First Hospital, Beijing, China; ^5^Department of Nursing, Peking University First Hospital, Beijing, China; ^6^School of Basic Medicine and Clinical Pharmacy, China Pharmaceutical University, Jiangsu, China; ^7^Institute of Clinical Pharmacology, Peking University, Beijing, China

**Keywords:** rational use of drugs, pharmaceutical interventions, older patients, potentially inappropriate medications, meta-analysis

## Abstract

**Introduction:**

Potentially inappropriate medications (PIMs) is a particular concern in older patients and is associated with negative health outcomes. As various interventions have been developed to manage it, we performed a systematic review and meta-analysis to evaluate the effect of pharmaceutical interventions on outcomes of PIMs in older patients.

**Methods:**

Meta-analysis of eligible randomized controlled trials (RCTs) was conducted to report the outcomes of pharmaceutical interventions in older patients searching from the databases of Cochrane Library, PubMed, Embase, Web of Science, Clinicaltrials.gov, SinoMed and Chinese Clinical Trial Registry (ChiCTR). The PRISMA guidelines were followed and the protocol was registered in PROSPERO (CRD42019134754). Cochrane bias risk assessment tool and the modified Jadad scale were used to assess the risk bias. RevMan software was used for data processing, analysis and graphical plotting.

**Results:**

Sixty-five thousand, nine hundred seventy-one patients in 14 RCTs were included. Of the primary outcomes, pharmaceutical interventions could significantly reduce the incidence of PIMs in older patients (OR = 0.51, 95% CI: 0.42, 0.62; *p* < 0.001), and the number of PIMs per person (MD = -0.41, 95%CI: −0.51, −0.31; *p* < 0.001), accompanying by a low heterogeneity. Subgroup analysis showed that the application of computer-based clinical decision support for pharmacological interventions could remarkably decrease the incidence of PIMs and two assessment tools were more effective. Of the secondary outcomes, the meta-analysis showed that pharmacological interventions could reduce the number of drugs used per person (MD = -0.94, 95%CI: −1.51, −0.36; *p* = 0.001) and 30-day readmission rate (OR = 0.58, 95%CI: 0.36, 0.92; *p* = 0.02), accompanying by a low heterogeneity. However, the pharmaceutical interventions demonstrated no significant improvement on all-cause mortality and the number of falls.

**Conclusion:**

Our findings supported the efficacy of pharmaceutical interventions to optimize the use and management of drugs in older patients.

**Systematic review registration:**

https://clinicaltrials.gov/, CRD42019134754.

## Introduction

The social and economic implications of older population are becoming increasingly apparent worldwide. The World Health Organization (WHO) has estimated that more than one in five individuals will be aged over 60 years by 2050, accounting for a total of 2 billion people in the world (1). Due to the prevalence of diverse diseases among older people, medications are commonly used to control the progression of diseases (2). In addition, the majority of older patients are treated by different therapeutic strategies. Therefore, concurrent treatment with polypharmacy is common among older people (3).

With the degradation of organs and physiological functions in older people, the pharmacokinetics and pharmacodynamics in the system could also change, thus, the risks of adverse drug reaction (ADR) and adverse drug event (ADE) could be elevated (4, 5), as well as increasing the rate of mortality (6) and the incidence of potentially inappropriate medications (PIMs). PIMs comprise a number of suboptimal prescribing practices, including inappropriate dose or duration of medication, drug–drug interaction, and drug-disease interaction (4). PIMs in older people are highly prevalent across a variety of healthcare settings, and are associated with the increased risk of ADEs, morbidity, mortality, health expenditure, healthcare utilization and frequent falling (7–10). It was estimated that 20–30% of all hospital admissions were related to prescription of drugs (11, 12), and up to 10% of all ADEs could be life-threatening or fatal (13).

Pharmaceutical intervention is the combination of modern pharmacology and clinical medicine, aiming to optimize the individualized drug therapy, improve the therapeutic effects of drugs, reduce the risk of the drug therapy, and ensure the safety and cost-effectiveness of the drug therapy (14). Pharmaceutical interventions could be performed specifically according to the epidemiology of PIMs and strategies, including specific health education and targeted interventions, which could substantially reduce the incidence of PIMs in older people (15).

However, current evidences vary substantially, and there are still debates on the influences of pharmaceutical interventions on outcomes of PIMs in older patients. Previous Cochrane systematic reviews (15, 16) could not draw robust conclusions from the evidences due to variability in design, interventions, outcomes and results. The most recent systematic review (17), which included all types of studies, suggests that PIM-setting-directed interventions should be developed to promote the wellbeing of the older patients through PIM reduction. However, it only reviewed and compared different interventions and outcomes, and did not conduct a meta-analysis. Therefore, we performed a systematic review and meta-analysis base on high-quality RCTs to evaluate and provide more reliable evidence of pharmaceutical interventions on PIMs in older patients.

## Methods

This systematic review and meta-analysis was conducted and reported in accordance with the Preferred Reporting Items for Systematic Reviews and Meta-Analysis (PRISMA) statement (18). It has been registered in the International Prospective Register of Systematic Reviews (CRD42019134754).

### Search strategy

The systematic search was undertaken in February 17, 2021, using the defined PICOS statement (population, intervention, comparison, outcomes, study type) to identify all relevant articles: Cochrane Library, PubMed, Embase, Web of Science, Clinicaltrials.gov, SinoMed and Chinese Clinical Trial Registry (ChiCTR) databases. Population was defined as older patients, which included the patients older than 65 years old or the studies clearly defined older patients. For the intervention, we focused on the pharmaceutical interventions on potentially inappropriate medications, including all kinds of strategies and tools/criterions. For outcome, we included studies reporting the rationally of medication use (incidence of PIMs, the number of drugs used and so on) and the prognosis of patients (mortality，falls and so on). RCT followed our PICOs framework was included. The terms used in the searching process included “aged” and “inappropriate medications” for English databases, and the corresponding Chinese terms were used in the Chinese databases. The references of the included studies were also reviewed to reduce the rate of missing data. The Appendix Table 1 listed as example of full search strings applicable to PubMed.

### Inclusion and exclusion criteria

The inclusion criteria were as follows: (1) RCTs; (2) subjects who aged ≥65 years old; (3) involvement of pharmaceutical intervention in the study group, and routine diagnosis and treatment in the control group; (4) outcome included the occurrence of PIMs.

The exclusion criteria were as follows: (1) unclear study design; (2) unclear interventions; (3) unclear outcomes; (4) duplicate publication; (5) unpublished studies; and (6) full-text could not be retrieved.

Compared with the protocol registered (CRD42019134754) in PROSPERO, since few studies reported the number of drugs used, it is difficult to determine polypharmacy, and the non-hospitalized patients such as outpatient and emergency department could not be ignored, the included population was expanded to all older patients over 65 years old. At the same time, intervention was usually not performed and completed by pharmacists independently, and it is impossible to evaluate the effect of pharmacist interventions alone, so it is expanded to all pharmaceutical interventions.

### Data extraction and quality assessment

Data were extracted using the predefined data extraction form by two investigators independently and cross-check was also performed. The disagreements were resolved by making discussion between the two investigators or involved the third investigator. The extracted data included: (1) general characteristics and data of the included studies, involving the first authors’ full-name, year of publication, country, study type, subjects’ age, and sample size; (2) indicators of study quality, such as the methods of randomization, allocation concealment, blinding, drop out, and loss to follow-up; (3) detailed measurement of intervention; and (4) indicators of clinical outcomes (PIMs-related clinical and economic outcomes, as well as humanistic outcomes, for example, health-related quality of life, patient satisfaction, medication adherence and so on).

Cochrane bias risk assessment tool (19) and the modified Jadad scale (20) were utilized for the assessment of the included studies. The Cochrane bias risk assessment tool covered the following 7 aspects: (1) randomization; (2) allocation concealment; (3) blinding to subjects and investigators; (4) blinding to evaluators; (5) completeness of outcome data; (6) selective report of data; and (7) other sources of bias. The modified Jadad scale evaluated the quality of the included studies from 4 aspects, such as randomization, allocation concealment, blinding, and drop out, and the assessment results were classified as “appropriate,” “unclear,” and “inappropriate,” with the corresponding scores of 2, 1, and 0, respectively. Studies with the total score of 4–7 points were considered as high-quality, and studies with the total score of 1–3 points were considered as low-quality.

### Statistical analysis

Review Manager 5.4 software was used for data processing. Heterogeneity was assessed by the *I*^2^ index and Q test (21). *I*^2^ ≤ 50% indicate low heterogeneity, and the fixed-effects model was used to pool the results. Otherwise, *I*^2^>50% represent substantial or considerable heterogeneity, and the random-effects model was utilized (22). For the pooled analysis of binary variables, odds ratio (OR) and corresponding 95% confidence interval (CI) were estimated. For the pooled analysis of continuous variables, mean difference (MD) and 95% CI were estimated. For outcome indicators that could not be analyzed by the meta-analysis, the descriptive analysis was applied.

## Results

### Search strategy

**Figure 1:**
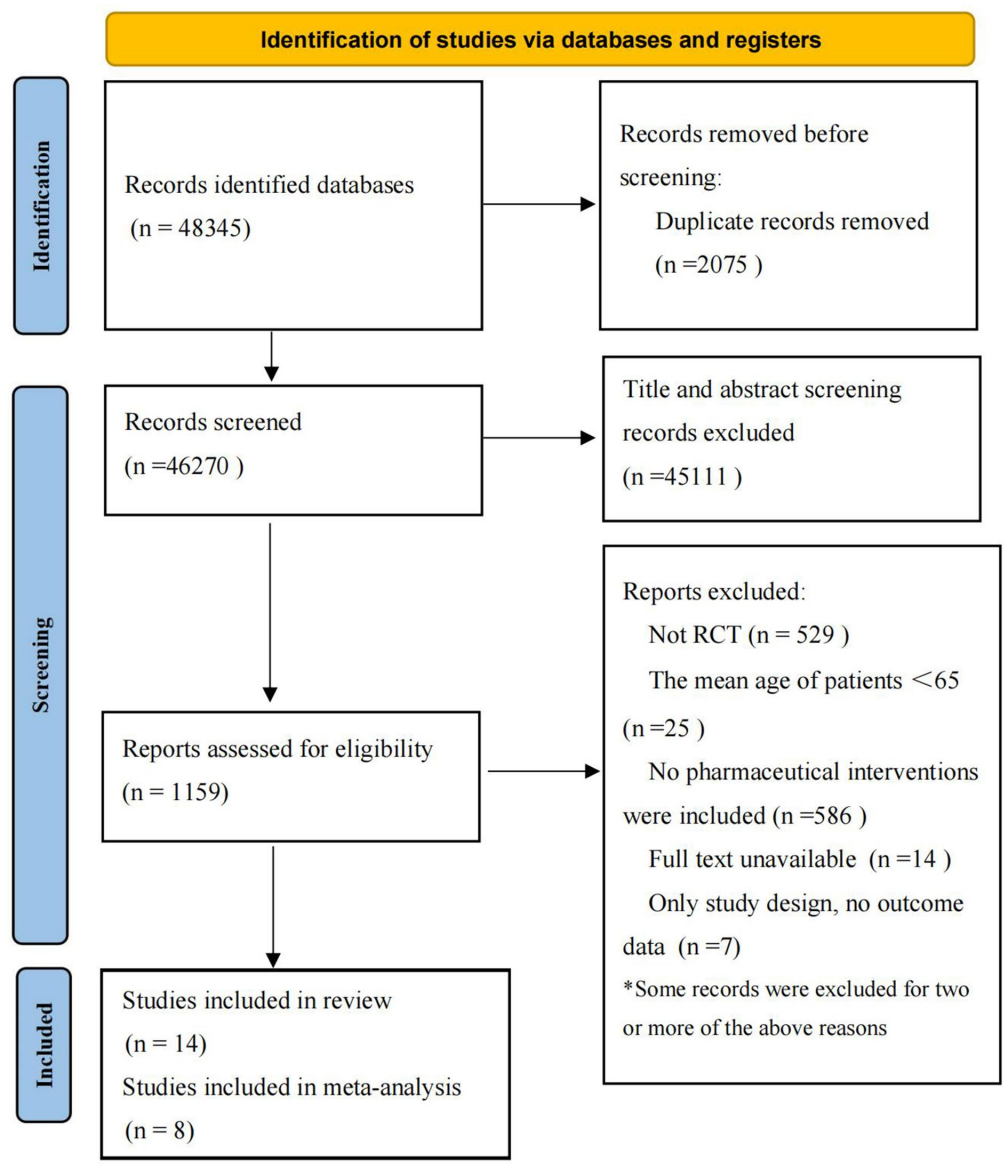
Process of study selection for RCTs.

Totally, 48,345 studies were retrieved by the initial screening. After further screening according to the inclusion and exclusion criteria, 14 RCTs were finally included in review 8 RCTs included in meta-analysis. The study flowchart is shown in Figure 1.

### General characteristics of the included studies

The detailed general characteristics of 14 RCTs are presented in Table 1.

**Table 1:** Characteristics of included trials.

First author, published year	Country	Patients	Age (year)	Sample size		Organization of intervention	Strategies of intervention	Tools for assessing PIM	CDSS	Outcomes	Follow-up (months)
				Intervention	control						
Schmader et al. (31)	America	Veterans	≥65	430	404	Multidisciplinary team	A, B	a,b	No	1,2,5	12
Ryan 2019 (32)	America	Patients with cancer	≥65	29	31	Pharmacists led	A, C, D, E	b	No	1,4	2
Martin et al. (28)	Canada	Community patients	≥65	248	241	Pharmacists led	F, G	b	Yes	1,6	6
Goedele 2019 (26)	Belgium	Nursing home patient	≥65	847	957	Multidisciplinary team	A, B	b,c	Yes	1	15
Cossette et al. (29)	Canada	Hospitalized patients	≥65	126	128	Multidisciplinary team	A, B	b,c	Yes	1,6,7,10,11,13	2.5
Veronica 2013 (34)	Sweden	Nursing home or community patients	≥75	182	187	Multidisciplinary team	A, D, E	d	No	1,6	2
Ulrika 2013 (35)	Sweden	Older patients	≥80	182	186	Multidisciplinary team	A, E, G	a,c	No	1,2	12
Van der Linden et al. (27)	Belgium	Hospitalized patients	≥65	32	29	Pharmacists led	A, B	d	No	1	2
Maria 2018 (36)	Sweden	Dementia or cognitive impairment patients	≥65	212	217	Multidisciplinary team	A, E	d	No	1,7,11,13	6
Dvora 2017 (25)	Israel	Nursing home patient	≥65	160	146	Pharmacists led	A, E	c	No	1,3,8,12	12
García et al. (24)	Spain	Nursing home patient	≥65	344	372	Pharmacists led	F, H	c	No	1,3,7,8,9,10	6
Patterson et al. (23)	Ireland	Nursing home patient	≥65	173	162	Pharmacists led	A, D, E	d	No	1,6	12
Allard et al. (30)	Canada	Community patients	>75	136	130	Multidisciplinary team	A, E	d	No	1,3	12
Marsha 2007 (33)	America	Older patients	≥65	29,840	29,840	Multidisciplinary team	A, E	d	Yes	1	12

Strategies of intervention: A, medication assessment; B, medication management; C, face-to-face communication with patients; D, recording of medications; E, communication of pharmacologists with doctors on medication strategy through discussion in meetings, face-to-face communication, telephone call, e-mail, and fax; F, dispatching materials of education to medical staff; G, providing health education for patients through paper materials or online videos; and H, providing training for medical staff. Tools for assessing PIM: a. MAI index, b. Beers criterion, c. STOPP/START criterion, d. the list of potentially inappropriate medications prepared by the Drug Commission. Outcomes:1. potentially inappropriate medications (PIMs), 2. MAI index, 3. the number of drugs used, 4. Vaccination coverage rates, 5. Incidence of adverse reactions, 6. Mortality rate, 7. Emergency attendance rate, 8. Average number of falls, 9. Average number of deliriums, 10. Length of stay, 11. Readmission rate, 12. Cost of medicines, 13. Readmission time.

The included studies were, respectively, performed in 7 countries, including 1 in Ireland (23), 1 in Spain (24), 1 in Israel (25), 2 in Belgium (26, 27), 3 in Canada (28–30), 3 in the United States (31–33), and 3 in Sweden (34–36).

6 of the 14 RCTs included the old patients from nursing home (24, 25, 29, 34) or community-dwelling (28, 30, 34), which is the main facility for long-term medication treatment of older patients. The subjects were veterans in 1 study (31), cancer patients in 1 study (32), and patients with dementia or cognitive dysfunctions in 1 study (36). Finally, 65,971 subjects, including 32,941 in the study group and 33,030 in the control group, were involved in the present meta-analysis.

Pharmaceutical intervention strategies are diverse and distinctive, with assessment and management of medication being the most common intervention.12 (23, 25–27, 29–40) of the 14 studies implemented similar interventions, for example, a team to provide strict surveillance and feedback for all medications. Interventions in 8 studies (23, 25, 30, 32–36) was to optimize medication use through clinical pharmacist-physician discussion in meetings, face-to-face, telephone call, e-mail communication. Regarding interventions for patients, medication education is provided through paper materials or online videos (28, 35) and face-to-face communication (32).

The tools for assessing PIM, included Beers’ criteria, screening tool of older person’s potentially inappropriate prescription (STOPP)/screening tool to alert doctors to the right treatment (START) criteria, and PIM list designed by the pharmaceutical council. In addition, 12 studies (23–25, 27, 28, 30–36) used one assessment tool, 2 studies (26, 29) utilized 2 assessment tools, 5 studies (26, 28, 29, 31, 32) employed the Beers’ criteria, 5 studies (24–26, 29, 35) used the STOPP/START criteria, and 6 studies (23, 27, 30, 33, 34, 36) utilized the PIM list designed by the pharmaceutical council.

From the 14 RCTs included in the study, a total of 13 outcomes were reported (the outcome column of Table 1), of which PIMs was the most widely evaluated and considered as the primary outcomes. All the 14 studies (23–36) evaluated PIMs, 4 studies (23, 28, 29, 34) reported all-cause mortality, 3 studies (24, 29, 36) reported all-cause emergent admission rate, 3 studies (24, 25, 30) reported the number of drugs used, 2 studies (24, 29) reported length of stay in hospital, 2 studies (29, 36) reported readmissions rate within 30 days of hospital discharge, 2 studies (24, 25) reported the number of falling events, 1 study (31) reported incidence of ADR, 1 study (25) reported cost of drugs, 1 study (24) reported the number of delirium episodes events and the number of visits to physician or nurse. Besides, 4 studies (26, 28, 29, 33) utilized the computer-based clinical decision support (CCDS) for intervention.

### Quality assessment

Quality assessment was performed for the methodology of included studies Appendix Figure 1 shows the results of quality assessment by Cochrane bias risk assessment tool, in which “+” indicates meeting the requirements, “-” represents no satisfaction of the requirements, and “?” denotes an unclear status. There were 2 studies (31, 34), in which all the items met the requirements. Appendix Figure 2 illustrates the percentage of each item for quality assessment of the included studies. All the 14 included studies (23–36) used randomization, without selective reporting of data. Allocation concealment was applied in 10 studies (23, 24, 28–32, 34–36), double-blinding in 6 studies (24, 31, 33–36), blinding to evaluators in 9 studies (23, 24, 28, 30, 31, 33–36), complete data in 2 studies (31, 34), and other sources of bias in 3 studies (23, 25, 36).

**Figure 2:**
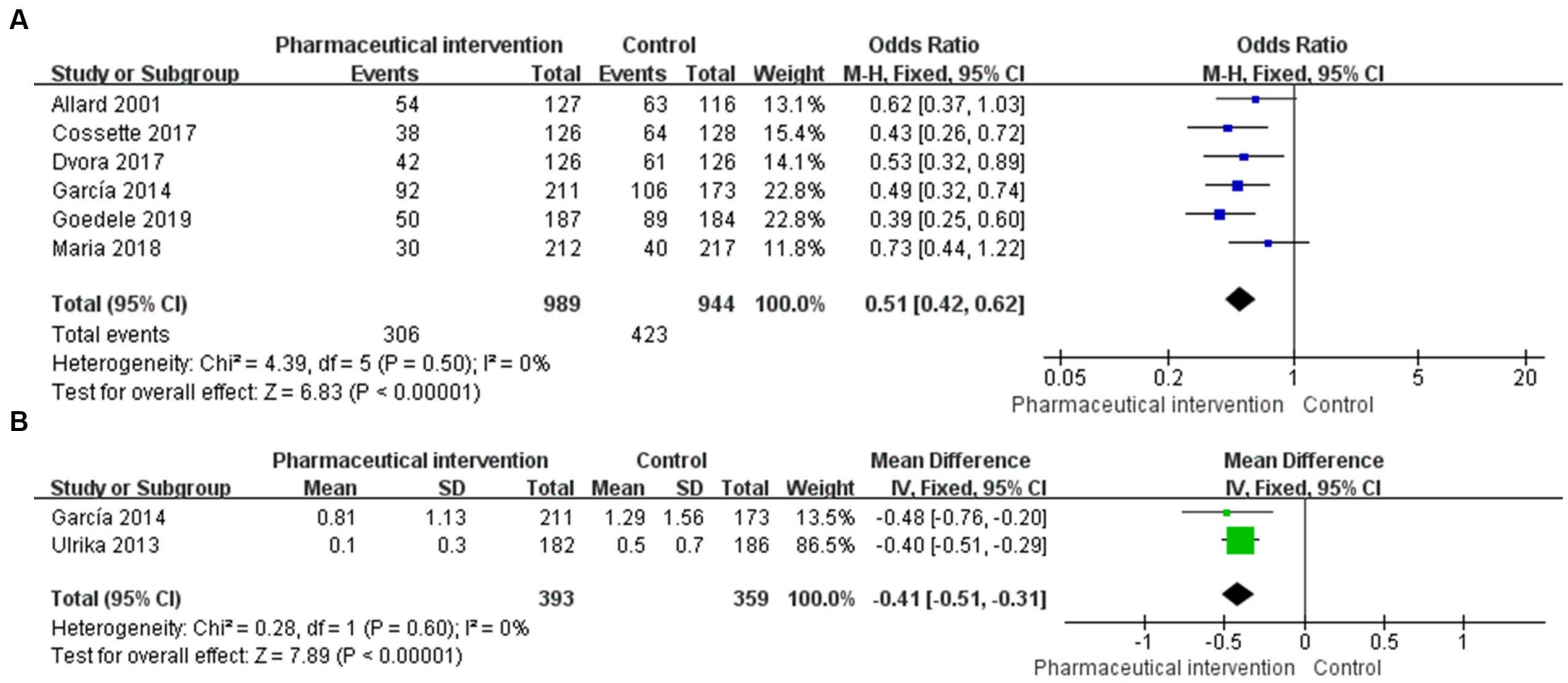
Forest plot for PIMs of eligible RCTs comparing pharmaceutical intervention with usual care **(A)** PIMs, **(B)** PIMs per person.

Base on the modified Jadad scale, 10 studies (23, 24, 28, 29, 31–36) had the quality score of 4–7 points, indicating that the risk of bias was low and the quality of studies was high. Moreover, 4 studies (25–27, 30) were found with the quality score of 1–3 points, demonstrating that the quality of studies was relatively low (Appendix Table 2).

### Primary outcomes

#### Incidence of PIMs

PIMs were reported in 14 studies, in which 6 studies (24–26, 29, 30, 36) that enrolled 1933 subjects (989 in the study group and 944 in the control group) reported the incidence of PIMs. The heterogeneity test showed a low heterogeneity among studies (*I*^2^ = 0%, *p* = 0.50), and thus, the fixed-effects model was used for the pooled analysis. The findings revealed that pharmaceutical intervention could significantly reduce the incidence of PIMs (OR = 0.51, 95%CI: 0.42, 0.62; *p* < 0.001; Figure 2A). Subgroup analysis was performed based on the application of CCDS, selection of assessment tools, and interventional strategies.

**Figure 3:**
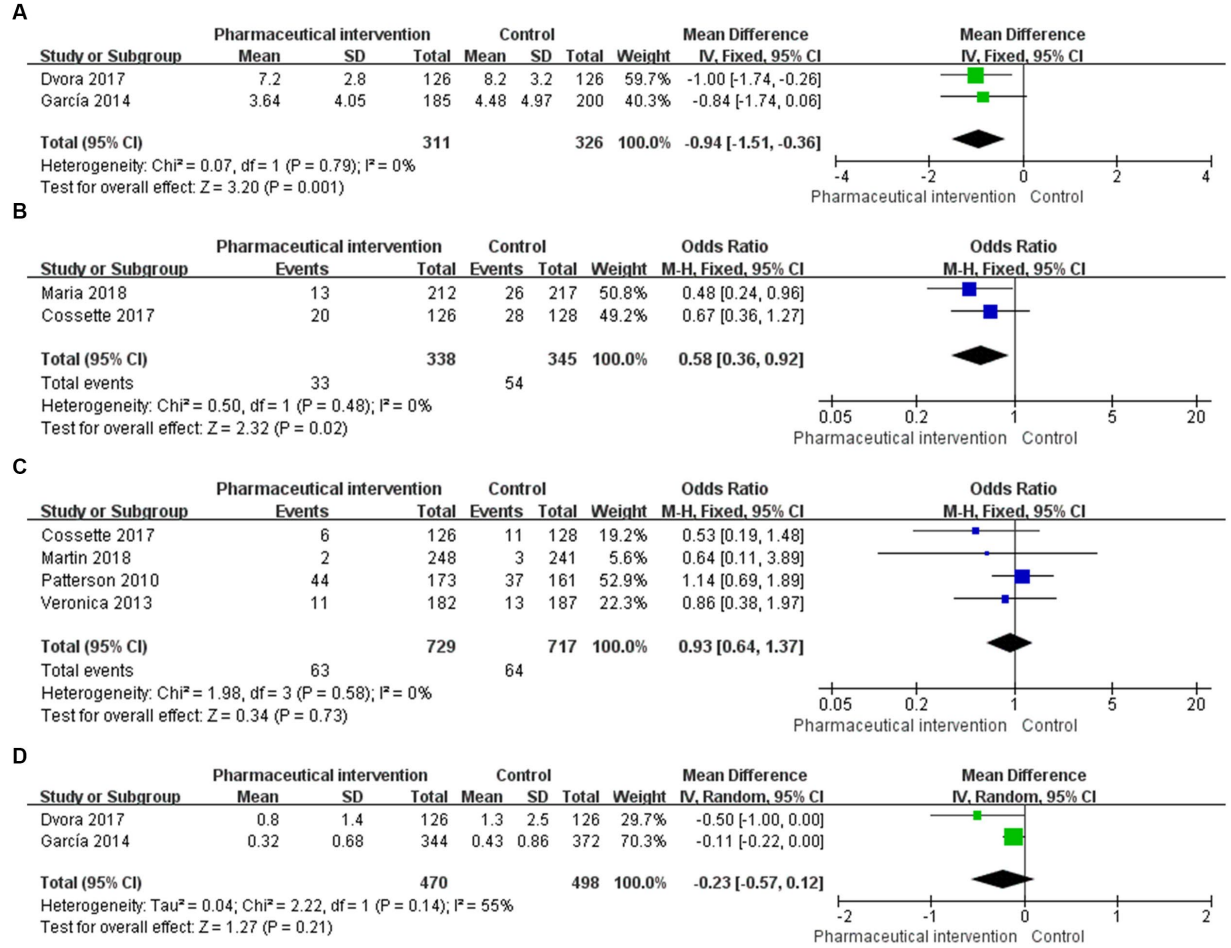
Forest plot for outcomes of eligible RCTs comparing pharmaceutical intervention with usual care **(A)** number of drugs used, **(B)** 30-days readmission, **(C)** all-cause mortality, **(D)** falling.

Of the 6 studies that reported the incidence of PIMs, 2 studies (26, 29) used the CCDS. The CCDS could automatically acquire the basic characteristics of patients, such as age, gender, body weight, comorbidities, medications, and examination results. In addition, the CCDS could send alerts when the medication changes, assisting the mediation assessment and pharmaceutical interventions. The comparison of the incidence of PIMs between the study group and control group that employed the CCDS showed that the difference was statistically significant (OR = 0.41, 95%CI: 0.29, 0.57; *p* < 0.001). The comparison of the incidence of PIMs between these two groups that did not employ the CCDS indicated that the difference was also statistically significant (OR = 0.57, 95%CI: 0.45, 0.73; *p* < 0.001). Although both subgroup comparisons achieved statistical significance, the pooled intervention effects of trials employing CCDS were superior to those without employing CCDS in terms of the magnitude of effect size (Appendix Figure 2A).

Of the 6 studies that reported the incidence of PIMs, 2 studies (26, 29) used the Beers’ criteria, 4 studies (24–26, 29) utilized the STOPP/START criteria, and the other studies employed the PIM list designed by the pharmaceutical council. Besides, 2 studies (26, 29) of the 6 studies used two assessment tools.

The comparison of the incidence of PIMs between the two groups that used one assessment tool revealed that the difference was statistically significant (OR = 0.57, 95%CI: 0.45, 0.73; *p* < 0.001). The comparison of the incidence of PIMs between the two groups that used two assessment tools showed that the difference was also statistically significant (OR = 0.41, 95%CI: 0.29, 0.57; *p* < 0.001). Although both subgroup comparisons achieved statistical significance, the pooled intervention effects of trials using two assessment tools were superior to those using single assessment tool in terms of the magnitude of effect size (Appendix Figure 2B).

#### Potentially inappropriate medications per person

Of the 14 studies that reported the incidence of PIMs, 2 studies (24, 35) reported PIMs per person, which totally included 752 subjects (393 in the study group and 359 in the control group). The heterogeneity test showed a low heterogeneity among the included studies (*I*^2^ = 0%, *p* = 0.60), and thus, the fixed-effects model was used for the pooled analysis. The findings demonstrated that pharmaceutical intervention could significantly reduce the incidence of PIMs per person in older people (MD = -0.41, 95%CI: −0.51, −0.31; *p* < 0.001; Figure 2B).

### Secondary outcomes

#### The number of drugs used per person

Among 3 studies that reported the number of drugs used, 2 studies (24, 25) that enrolled 637 patients (311 in the study group and 326 in the control group) reported the number of drugs used per person. The heterogeneity test showed a low heterogeneity among the included studies (*I*^2^ = 0%, *p* = 0.79), and the fixed-effects model was used for the pooled analysis. The results showed that pharmaceutical intervention could significantly reduce the number of drugs used per person in older people (MD = -0.94, 95%CI: −1.51, −0.36, *p* = 0.001; Figure 3A).

#### 30-day readmission rate

In 2 studies (29, 36) that reported the 30-day readmission rate, 683 subjects were enrolled, including 338 in the study group and 345 in the control group. Heterogeneity test showed a low heterogeneity among the eligible studies (*I*^2^ = 0%, *p* = 0.48), and the fixed-effects model was used for the pooled analysis. The findings revealed that the pharmaceutical intervention could significantly reduce the 30-day readmission rate (OR = 0.58, 95%CI: 0.36, 0.92; *p* = 0.02; Figure 3B).

#### All-cause mortality

There were 4 studies (23, 28, 29, 34) that reported the all-cause mortality and enrolled 1,446 subjects, of whom 729 and 717 subjects were in the study group and control group, respectively. Heterogeneity test showed a low heterogeneity among the included studies (*I*^2^ = 0%, *p* = 0.58), and the fixed-effects model was used for the pooled analysis. The results revealed that the effects of pharmaceutical intervention on the all-cause mortality were not significantly different between the two groups (OR = 0.93, 95%CI: 0.64, 1.37; *p* = 0.73; Figure 3C).

#### Falls

In 2 studies (24, 25) that reported the average number of falls, 968 subjects were enrolled, including 470 and 498 subjects in the study group and control group, respectively. Heterogeneity test showed a moderate heterogeneity among the eligible studies (*I*^2^ = 55%, *p* = 0.14), and the random-effects model was used for the pooled analysis. The findings showed that the effects of pharmaceutical intervention on the average number of falls were not significantly different between the two groups (MD = -0.23, 95%CI: −0.57, 0.12; *p* = 0.21; Figure 3D).

## Discussion

The present study systematically reviewed 14 RCTs to compare the effects of pharmaceutical interventions on older people. The results indicated that the incidence of PIMs, the number of PIMs per person, the number of drugs used, and 30-day readmission rate were significantly lower in the pharmaceutical intervention group, and the subgroup analysis showed that the application of the CCDS and assessment tools, such as Beers’ and STOPP criteria, could markedly reduce the incidence of PIMs. It is noteworthy that the current study further comprehensively evaluated the effect of pharmaceutical intervention on potentially inappropriate medications in older patients by including high-quality RCTs and integrating intervention effects through meta-analysis.

PIMs are associated with risks that are greater than their potential benefits, and older patients are at a greater risk of ADEs from PIMs. Reducing the incidence of PIMs will be significant to reduce the incidence of ADEs and improve the prognosis of older patients. There are a variety of intervention strategies and methods that can effectively reduce the incidence of the PIMs. Subgroup analysis showed that the application of CCDS could reduce the incidence of the PIMs, which is consistent with the result of a previous study (37). The advantage of CDSS is not only associated with the efficient and accurate identification and reduction of PIMs, but also with the ability to change the prescribing behavior of physicians (38). In the present study, further subgroup analysis suggested that using single assessment tool may not be comprehensive in assessing PIMs, and the multiple assessment tools achieved a more desirable intervention result. This finding is consistent with the conclusion of Kurczewska-Michalak et al. (41) that no “gold standard” is identifiable and advisable, and the complexity, applicability and usability of tools needs to be considered. The complementarity of the different tools was also confirmed by Lisowska et al. (42), the PILA tool that included STOPP/START v.2 and Amsterdam tool identified the highest number of PIMs and achieved the most comprehensive assessment of pharmacotherapy appropriateness in geriatric patients. Overall, CDSS combining different prescription indicators should be considered as an important tool to optimize drug prescription for older patients.

Pharmaceutical interventions could significantly reduce the number of drugs used per person and 30-day readmission rate. Deprescribing is the process of systematically reviewing a patient’s medications and discontinuing drugs in instances, in which existing or potential harms outweigh existing or potential benefits within the context of an individual patient’s care goals, current level of functioning, life expectancy, values, and preferences (39, 40). A growing body of evidence related to the adverse effects of polypharmacy on older patients supported the need for deprescribing (43), and the number of drugs that a patient is taking is the most important predictor of ADEs (44). Therefore, the number of drugs used per person and 30-day readmission rate reflect the efficacy and safety of drug therapy, as well as being important indicators for testing the effects of pharmaceutical interventions. Additionally, regarding the economic endpoint, although only one study (25) reported the cost of drugs, pharmaceutical interventions could effectively reduce the cost of drugs in the study group compared with that in the control group. Economic systematic reviews (45) focusing on polypharmacy have expressed the same view, with interventions generally associated with a reduction in medication expenditure. Available evidence suggests that the potential benefits of interventions to optimize medication use outweigh the costs of their implementation, and the results of the included cost–benefit analysis studies (46–48) showed a net benefit that was null or positive.

The all-cause mortality and average number of falls were both lower in the study group than those in the control group, however, the differences were not statistically significant. The mortality rates were consistent with those reported previously (15). This can be related to contribution of other factors affecting mortality, of which disease progression is noteworthy. However, there are still some drug-related factors that require clinicians’ attention (49–51). Several studies (52, 53) have shown that targeted pharmacological interventions for fall risk, including withdrawal of potential fall-risk-increasing drugs (FRIDs), pharmacist-conducted clinical medication review, and computerized drug alerts, were effective in reducing fall risk. However, tools for assessing PIM, such as Beers’ and STOPP criteria, do not concentrate on FRIDs. Therefore, in the comprehensive pharmacological intervention, the management of FRIDs still needs to be improved, and it is suggested to reduce the fall risk in older patients with polypharmacy.

Because no “gold standard” has been identified, multiple interventions toward PIMs are advised, so the diversity of interventions included and analyzes in our meta-analysis is critical. Compared with the a scoping review of available interventions published in 2012 (41), relevant interventions, including prescription deprescribing, CDSS, medication therapy management (MTM) and so on, were all included in this meta-analysis. Regarding the setting and supporter of pharmaceutical interventions, the most common setting is primary healthcare team. Additionally, some interventions were provided at community or hospital pharmacies, in the form of pharmacists alone or in cooperation with a physician or nurse. In summary, the following steps involved pharmacists are the key to appropriate medications in older patients: patients’ evaluation and data collection, medication review, being agree with patients on treatment objectives, prescription decision, communication and obtaining patient agreement, medication dispensing, medication usage, monitoring and assessment (41, 54).

Totally, 14 eligible RCTs were involved in the present study, which were mainly accompanied by a relatively high-quality. However, the limitations of this study were summarized as follows: (1) outcomes in the included studies varied, and only relatively few studies reported the same outcomes; (2) the data of the included studies were reported inconsistently, and thus, some outcomes could not be pooled; (3) only one study reported the cost of drugs; (4) difference in ethnicity, language, educational level, places for interventions and follow-up time among the subjects and studies all might influence the outcomes. (5) the possibility of missing studies not included in the databases we used and the articles published in other langue not being included in our analysis, (6) the risk of bias cannot be excluded, although most heterogeneity is acceptable. Therefore, more rigorously designed multi-center RCTs with larger sample size and longer follow-up with high-quality are needed to further validate the findings of the present study.

## Conclusion

In summary, pharmaceutical interventions may improve the prognosis of older patients via reducing the incidence of PIMs, the number of PIMs per person, the number of drugs used, and 30-day readmission rate. Our findings supported the efficacy of pharmaceutical interventions to optimize the use and management of drugs for older patients.

## Data availability statement

The original contributions presented in the study are included in the article/Supplementary material, further inquiries can be directed to the corresponding author.

## Author contributions

CY and ZhY: literature search. LR, ZoY, and LL: screening of the search results. ZS, LR, and ZhY: risk of bias assessment. LR and LL: interpretation of the results. ZS, ZX, TZ, and ZoY: drafting and revising the article. ZS, LR, ZX, and LH: final approval of the version to be published. All authors contributed to the article and approved the submitted version.

## Funding

This study was supported by National Key R&D Program of China (2020YFC2008804) and grants National High-Level Hospital Clinical Research Funding (Scientific Research Seed Fund of Peking University First Hospital, no.2022SF87).

## Conflict of interest

The authors declare that the research was conducted in the absence of any commercial or financial relationships that could be construed as a potential conflict of interest.

## Publisher’s note

All claims expressed in this article are solely those of the authors and do not necessarily represent those of their affiliated organizations, or those of the publisher, the editors and the reviewers. Any product that may be evaluated in this article, or claim that may be made by its manufacturer, is not guaranteed or endorsed by the publisher.
