# Supplementary material for: The effects of pharmaceutical interventions on potentially inappropriate medications in older patients: a systematic review and meta-analysis
Source: Front Public Health. 2023 Jul 11;11:1154048. doi: 10.3389/fpubh.2023.1154048 (PMC10368444; doi:10.3389/fpubh.2023.1154048)
Supplement: Supplementary file 1 [file Table_1.DOCX]

**Appendix Table 1. The search strategy and details of Pubmed**

| Database | Search | Query | Results |
| --- | --- | --- | --- |
| Pubmed | 1 | ((((("Aged"[Mesh]) OR (Elderly[Title/Abstract]))) | 3,465,395 |
|  | 2 | ((("Potentially Inappropriate Medication List"[Mesh]) OR (((((((((((((((((((((((((((((((((((((((((((((((PIM List[Title/Abstract]) OR (PIM Lists[Title/Abstract])) OR (Potentially Inappropriate Medications[Title/Abstract])) OR (Inappropriate Medication, Potentially[Title/Abstract])) OR (Inappropriate Medications, Potentially[Title/Abstract])) OR (Medication, Potentially Inappropriate[Title/Abstract])) OR (Medications, Potentially Inappropriate[Title/Abstract])) OR (Potentially Inappropriate Medication[Title/Abstract])) OR (Beers Criteria[Title/Abstract])) OR (Beers Potentially Inappropriate Medications[Title/Abstract])) OR (de Beers Criteria[Title/Abstract])) OR (Beers Criteria, de[Title/Abstract])) OR (STOPP (Screening Tool of Older Person's Potentially Inappropriate Prescriptions)[Title/Abstract])) OR (STOPPs (Screening Tool of Older Person's Potentially Inappropriate Prescriptions)[Title/Abstract])) OR (STOPP[Title/Abstract])) OR (STOPP START Criteria[Title/Abstract])) OR (Criteria, STOPP START[Title/Abstract])) OR (Criterias, STOPP START[Title/Abstract])) OR (START Criteria, STOPP[Title/Abstract])) OR (START Criterias, STOPP[Title/Abstract])) OR (STOPP START Criterias[Title/Abstract])) OR (Medication Appropriateness Index[Title/Abstract])) OR (Appropriateness Index, Medication[Title/Abstract])) OR (Appropriateness Indices, Medication[Title/Abstract])) OR (Index, Medication Appropriateness[Title/Abstract])) OR (Indices, Medication Appropriateness[Title/Abstract])) OR (Medication Appropriateness Indices[Title/Abstract])) OR (Inappropriate Prescribing[Title/Abstract])) OR (Medication Errors[Title/Abstract])) OR (Inappropriate Prescribings[Title/Abstract])) OR (Prescribing, Inappropriate[Title/Abstract])) OR (Prescribings, Inappropriate[Title/Abstract])) OR (Inappropriate Prescriptions[Title/Abstract])) OR (Inappropriate Prescription[Title/Abstract])) OR (Prescription, Inappropriate[Title/Abstract])) OR (Prescriptions, Inappropriate[Title/Abstract])) OR (Over Prescribing[Title/Abstract])) OR (Over Prescribings[Title/Abstract])) OR (Prescribing, Over[Title/Abstract])) OR (Prescribings, Over[Title/Abstract])) OR (Drug Prescriptions[Title/Abstract])) OR (Drug Prescription[Title/Abstract])) OR (Drug Prescribing[Title/Abstract])) OR (Drug Prescribings[Title/Abstract])) OR (Prescribing, Drug[Title/Abstract])) OR (Prescribings, Drug[Title/Abstract])))) | 14,903 |
|  | 3 | (1000/1/1:2021/2/17[pdat])) | 32,388,337 |
|  | 4 | #1 AND #2 | 5,098 |
|  | 5 | #3 AND #4 | 4,773 |
