# Supplementary material for: The effects of pharmaceutical interventions on potentially inappropriate medications in older patients: a systematic review and meta-analysis
Source: Front Public Health. 2023 Jul 11;11:1154048. doi: 10.3389/fpubh.2023.1154048 (PMC10368444; doi:10.3389/fpubh.2023.1154048)
Supplement: Supplementary file 2 [file Table_2.DOCX]

**E-Table 3. The Evaluation of the Methodological Quality of Included Studies by the modified Jadad scale**

| **first author，published year** | **description of randomization** | **description of allocation concealment** | **blinding description** | **description of withdrawals and dropouts** | **Jadad score** |
| --- | --- | --- | --- | --- | --- |
| Schmader 2004[31] | 2 | 2 | 2 | 0 | 6 |
| Ryan 2019[32] | 2 | 2 | 0 | 1 | 5 |
| Martin 2018[28] | 2 | 2 | 2 | 1 | 7 |
| Goedele 2019[26] | 1 | 1 | 0 | 0 | 2 |
| Cossette 2017[29] | 2 | 2 | 0 | 1 | 5 |
| Veronica 2013[34] | 2 | 2 | 2 | 0 | 6 |
| Ulrika 2013[35] | 2 | 2 | 2 | 1 | 7 |
| Van 2019[27] | 1 | 1 | 0 | 0 | 2 |
| Maria 2018[36] | 2 | 2 | 2 | 1 | 7 |
| Dvora 2017[25] | 1 | 1 | 0 | 1 | 3 |
| García 2014[24] | 2 | 2 | 2 | 1 | 7 |
| Patterson 2010[23] | 2 | 2 | 0 | 1 | 5 |
| Allard 2001[30] | 1 | 1 | 0 | 1 | 3 |
| Marsha 2007[33] | 2 | 2 | 2 | 1 | 7 |
