# Supplementary figures and images for: The effects of pharmaceutical interventions on potentially inappropriate medications in older patients: a systematic review and meta-analysis
Source: Front Public Health. 2023 Jul 11;11:1154048. doi: 10.3389/fpubh.2023.1154048 (PMC10368444; doi:10.3389/fpubh.2023.1154048)

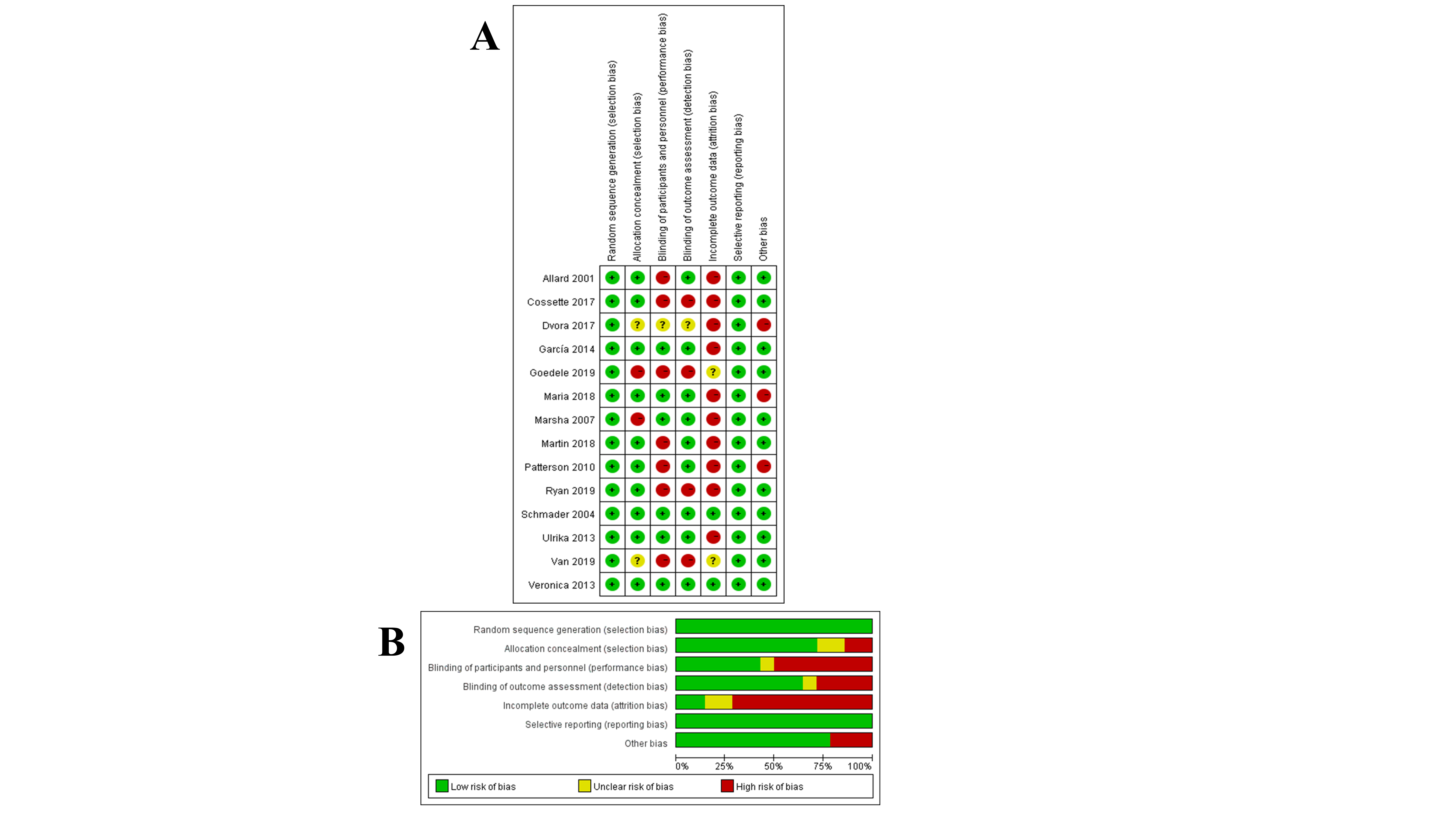

Supplement: Supplementary file 3 [file Image_1.PNG]

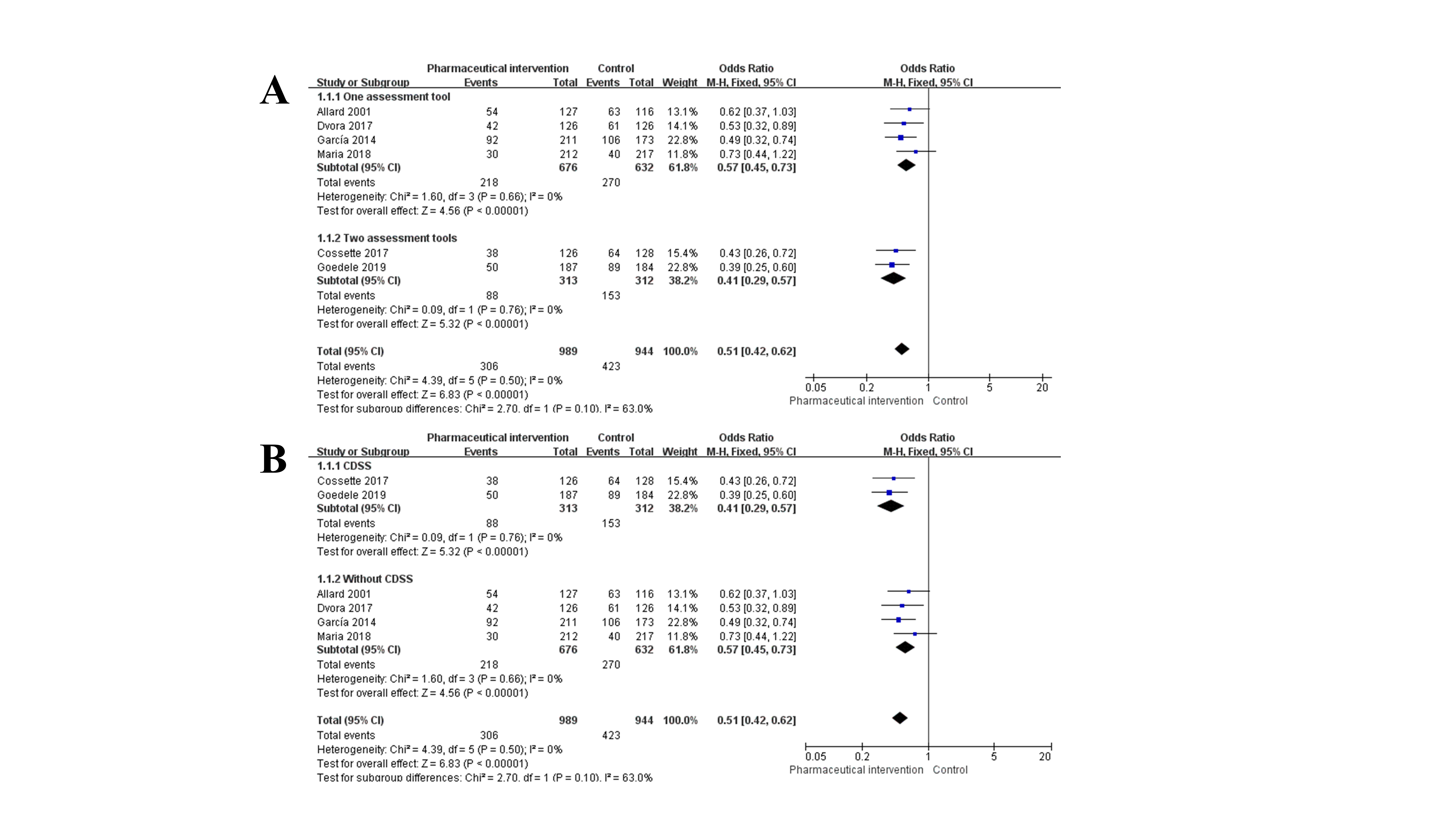

Supplement: Supplementary file 4 [file Image_2.PNG]
